# Disproportionality analysis of satralizumab in FDA adverse event reporting system and Japanese adverse drug event report: a pharmacovigilance study

**DOI:** 10.3389/fimmu.2025.1647306

**Published:** 2026-01-14

**Authors:** Linhai Zhang, Rong Yan, Zucai Xu

**Affiliations:** 1 Department of Neurology, Affiliated Hospital of Zunyi Medical University, Guizhou, China; 2 Key Laboratory of Brain Function and Brain Disease Prevention and Treatment of Guizhou Province, Zunyi, China

**Keywords:** FAERS, adverse events, NMOSD, satralizumab, pharmacoepidemiology

## Abstract

**Background:**

To date, no post-marketing safety studies of satralizumab have been conducted. This study aims to evaluate the post-marketing safety profile of satralizumab using data from the U.S. Food and Drug Administration (FDA) Adverse Event Reporting System (FAERS) and Japanese Adverse Drug Event Report (JADER) databases.

**Methods:**

We extracted all adverse event (AE) reports related to satralizumab from the FAERS database covering the period from Q1–2023 to Q4 2024. Disproportionality analyses were conducted for AEs with at least four reports listing satralizumab as the primary suspect (PS), using Reporting Odds Ratio (ROR), Proportional Reporting Ratio (PRR), Bayesian Confidence Propagation Neural Network (BCPNN), and Multi-item Gamma Poisson Shrinker (MGPS), along with their corresponding 95% confidence intervals (CI). Non-parametric tests were applied to compare differences between serious and non-serious outcomes.

**Results:**

Since its approval, a total of 2,527 AEs related to satralizumab have been reported in the FAERS database, involving 931 individual patients. Among them, 355 patients experienced serious outcomes, including 389 fatal cases. The most frequently reported AEs were infection-related, such as urinary tract infection (n = 72), infectious pneumonia (n = 44), COVID-19 (n = 43), sepsis (n = 23), and cellulitis (n = 17). In addition, several unexpected AEs were identified, including cerebral infarction and malignancies. Compared with patients experiencing non-serious outcomes, male patients were more likely to develop serious outcomes, which also tended to occur earlier in the treatment course. Furthermore, the standard usage (120 mg once every 2 weeks for three doses at Weeks 0, 2, and 4, followed by 120 mg once every 4 weeks) was associated with a relatively favorable safety profile.

**Conclusion:**

The adverse risks associated with satralizumab are notable. Our findings provide evidence to support risk assessment in clinical practice; however, high-quality clinical studies are still needed to validate these results and to further explore the long-term safety and efficacy of the drug.

## 1 Introduction

Satralizumab is a monoclonal antibody that inhibits the production of autoantibodies by blocking the interleukin-6 (IL-6) receptor. It was approved by the FDA in August 2020 and is currently used in the United States, the European Union, and other regions for the treatment of neuromyelitis optica spectrum disorder (NMOSD) in patients who are seropositive for aquaporin-4 (AQP4) antibodies (1).

In two pivotal clinical trials—SAkuraSky and SAkuraStar—with a median treatment duration of approximately four years, the incidence of AEs was comparable between the satralizumab and placebo groups. Most AEs were of mild to moderate severity, and no treatment-related fatal events were reported (2). The most frequently observed AEs during treatment included upper respiratory tract infections and urinary tract infections. Overall, satralizumab demonstrated a favorable safety and tolerability profile, both as monotherapy and as an add-on therapy.

To date, however, post-marketing safety data beyond large-scale clinical trials remain limited. The strict inclusion and exclusion criteria in randomized controlled trials often result in relatively small and homogeneous patient populations, which may introduce bias in the assessment of both therapeutic efficacy and adverse risks (3).

Spontaneous reporting systems have emerged as a valuable and convenient source of real-world data for post-marketing pharmacovigilance (4). Despite inherent limitations—such as underreporting, reporting bias, and variability in data quality—these systems play a crucial role in monitoring drug safety and identifying novel AE signals in the general population.

## 2 Methods

### 2.1 Data acquisition and processing

We integrated data from both the FAERS and JADER databases to analyze reported AEs associated with satralizumab. The search terms included “SATRALIZUMAB,” “ENSPRYNG SATRALIZUMAB,” “ENSPRYNG,” “SATRALIZUMAB MWGE,” and the Japanese term “サトラリズマブ.”

Duplicate reports were removed in accordance with the FDA’s recommended deduplication method, which retains only the most recent and complete version of each case. Specifically, duplicate entries were identified based on the unique CASEID and FDA_DT, and only the latest version of each report was preserved (5, 6).

### 2.2 Data algorithms

We utilized both Frequentist and Bayesian statistical methods to detect drug safety signals. Frequentist statistics included the ROR, PRR, BCPNN, and MGPS (7, 8). The relevant algorithms are detailed in **Supplementary Material**.

### 2.3 Signal prioritization

The FDA classifies AEs cases as either serious or non-serious. Serious cases include events such as death, hospitalization, disability, or life-threatening conditions. In our analysis, AEs were further categorized into four classes based on predictability: Expected AEs: Events that are anticipated based on the pharmacological mechanism of satralizumab or those previously reported in clinical trials; Disease-related AEs: Events attributable to the underlying disease (NMOSD) itself, which inherently poses a risk—for example, visual impairment, myelitis, or muscle weakness following NMOSD; Comorbidity-related AEs: Events associated with coexisting conditions or concomitant medications. For instance, NMOSD patients receiving baseline glucocorticoid therapy may be at increased risk of osteoporosis or fractures; Unexpected AEs: Previously unknown or unpredictable events (9).

### 2.4 Statistical analysis

To improve the consistency and robustness of our findings, we employed four disproportionality analysis methods: ROR, PRR, IC, and EBGM. All AEs with at least four reports listing satralizumab as the primary suspect were included, and the corresponding 95% CIs were calculated. Further details are provided in the **Supplementary Materials**. All statistical analyses were conducted using R software (version 4.4.2).

## 3 Results

### 3.1 Clinical characteristics

Over the four years since satralizumab was approved, a total of 2,527 satralizumab-related AEs were reported in the FAERS database, involving 931 patients—an average of 2.7 AEs per patient. Among these, 355 patients experienced serious AEs, including 38 deaths. The number of reported cases increased steadily over time, from 4 in 2020 to 398 in 2024. Age data were available for 611 patients, with a mean age of 47.6 ± 22.8 years. Of the 931 patients, 763 (82.0%) were female, 119 (12.8%) were male, and the sex was unknown in 49 cases (5.3%). AEs were reported by healthcare professionals in 611 cases (65.7%) and by consumers in 316 cases (33.9%) (**Table 1**).

**Table 1:** Characteristics of the patients for satralizumab.

Information	Overall (N = 931)
Sex	
F	763 (82.0%)
M	119 (12.8%)
Missing	49 (5.3%)
Age	
<18	85 (9.1%)
18~64.9	367 (39.4%)
65~85	157 (16.9%)
>85	2 (0.2%)
Missing	320 (34.4%)
Reporting year	
2020	4
2021	79
2022	166
2023	284
2024	398
Reporter	
Physician	523 (56.2%)
Consumer	316 (33.9%)
Health professor	51 (5.5%)
Pharmacist	37 (4.0%)
Missing	4 (0.4%)
Reporting country	
Japan	411 (44.1%)
United states	353 (37.9%)
China	31 (3.3%)
Canada	29 (3.1%)
Other	107 (11.5%)
Report outcome	
Death	38
Serious outcome: death, hospitalization, disability, or life-threatening.	355
Non-serious outcome:	576
Time to onset	
N (Missing)	332 (599)
Mean (SD)	210.66 (283.43)
Median (Q1, Q3)	(28.0, 284.5)
Min, Max	1, 1989

Among the AEs reported at least four times, a total of 50 distinct AE types were identified. The most frequently reported AEs included urinary tract infection (n = 72, 12.79%, mean time-to-onset: 234.7 days), infectious pneumonia (n = 44, 7.82%, 178.63 days), COVID-19 (n = 43, 7.64%, 211.74 days), hypoesthesia (n = 24, 4.26%, 56.6 days), sepsis (n = 23, 4.09%, 182.38 days), cellulitis (n = 17, 3.02%, 119.36 days), muscular weakness (n = 16, 2.84%, 238.5 days), abnormal liver function (n = 15, 2.66%, 74.85 days), and lymphocyte count decreased (n = 15, 2.66%, 196.71 days) (**Supplementary Table 3**). Baseline characteristics of patients who experienced fatal outcomes are summarized in **Table 2**.

**Table 2:** Characterization of deaths cases for satralizumab.

Information	Death cases (N = 38)
Sex	
Male	12
Female	25
Missing	1
Age	
<18	5
18-65	11
>65	13
Reporter	
Physician	31
Consumer	4
Health professor	2
Pharmacist	1
System organ class (Top5)	
Infections and Infestations	26
General disorders and Administration site conditions	12
Neoplasms benign, malignant and unspecified (incl cysts and polyps)	9
Respiratory, thoracic and mediastinal disorders	8
Investigations	7
Preferred term (see more in Supplementary Material)	
Infection ^a^	28
Death ^b^	8
Neoplastic diseases ^c^	6
Time to onset ^d^	
N (Missing)	19 (19)
Mean (SD)	181.68 (168.49)
Median (Q1, Q3)	109 (70.0, 286.5)
Min, max	7, 630

a . Includes infections (n = 3), urinary tract infections (n = 5), pulmonary infections (n = 4), COVID-19 (n = 5), sepsis (n = 3), septic shock (n = 3), encephalitis (n = 1), cystitis (n = 1), atypical mycobacterial infection (n = 1), osteomyelitis (n = 1), and nasopharyngitis (n = 1).

b . Includes death (n = 7) and brain death (n = 1).

c . Includes pulmonary tumor, bladder cancer, leukemia, and intraductal papillary mucinous neoplasm.

d . Time from treatment initiation to death.

### 3.2 Signal detection by sex

Given the female predominance in the epidemiology of NMOSD, the number of AEs reported in females was significantly higher than in males. However, signal detection analysis revealed that certain AEs were exclusively observed in male patients, including cerebral infarction, renal impairment, and decreased mobility, which were not reported among female cases. These sex-specific AE distributions are illustrated using volcano plots (**Supplementary Figures 1**, **2**).

### 3.3 Comparison between serious and non-serious outcomes

A comparative analysis between patients with serious and non-serious outcomes revealed a significant difference in sex distribution (*p* = 0.018), with male patients being at a higher risk of experiencing serious outcomes than female patients.

Among patients with known dosing regimens, those receiving either every-2-week (q2w) or every-4-week (q4w) administration schedules had an increased risk of serious outcomes compared to the standard regimen[OR: 2.61 (95% CI: 1.07–6.56) for q2w; OR: 1.48 (95% CI: 0.69–3.23) for q4w].

**Figure 1:**
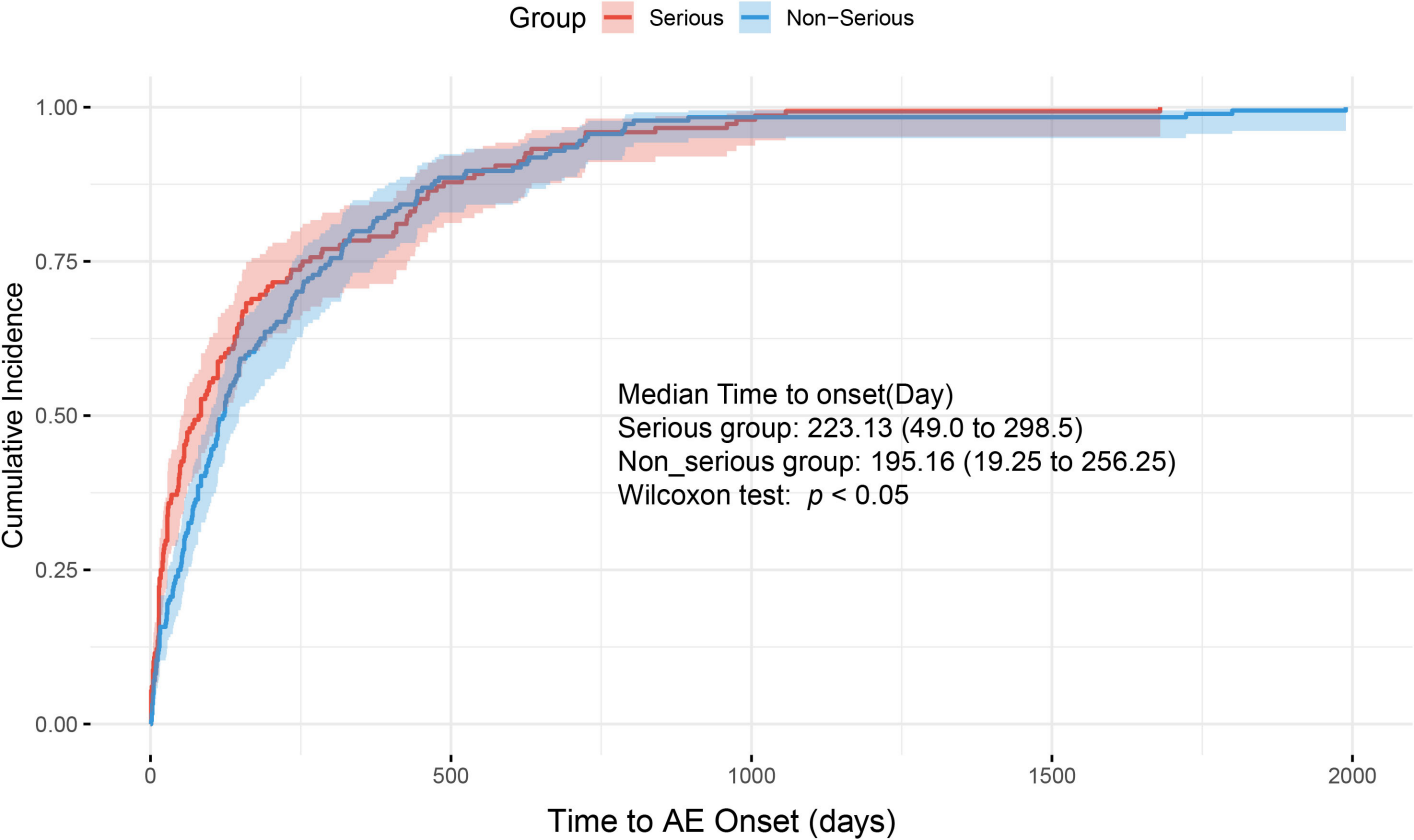
Cumulative incidence curves of time to adverse event onset in patients with serious versus non-serious outcomes.

Time-to-onset also differed significantly between groups (*p* < 0.05), with serious outcomes more likely to occur earlier in the treatment course (**Figure 1**). In contrast, no statistically significant differences were observed in age (*p* = 0.729) or body weight (*p* = 0.06).

Certain AEs were more frequently associated with serious outcomes, including urinary tract infection (*p* < 0.001), pneumonia (*p* = 0.006), cellulitis (*p* = 0.013), and generalized infection (*p* = 0.022).

### 3.4 Signal detection in the JADER database

The JADER database was used as a comparator to validate safety signals. Among the preferred terms (PTs) with more than four reported cases, the overall AE profile was largely consistent with that observed in the FAERS database. However, several PTs were identified exclusively in JADER, including anemia, Escherichia coli urinary tract infection, and pancytopenia, which were not detected in FAERS (**Supplementary Table 4**).

## 4 Discussion

NMOSD is a rare autoimmune inflammatory disease primarily characterized by episodes of optic neuritis and longitudinally extensive transverse myelitis. A major breakthrough in understanding its pathogenesis was the discovery of a specific autoantibody, NMO-IgG, which targets AQP4, a water channel protein highly expressed on astrocyte foot processes (10). The clinical manifestations of NMOSD depend on the anatomical location of AQP4-IgG–mediated astrocyte injury and associated inflammatory responses, commonly presenting as vision loss, motor weakness, and intractable hiccups (11).

The heterogeneity in the pathophysiological mechanisms of NMOSD has led to the development of several targeted therapies. These include B-cell–directed monoclonal antibodies such as ublituximab and rituximab (anti-CD20), IL-6 inhibitors such as tocilizumab, and complement pathway inhibitors such as eculizumab (12). IL-6 plays a critical role in NMOSD by promoting the differentiation of naïve T cells into pro-inflammatory Th17 cells and driving B cell maturation into AQP4-IgG–secreting plasma cells (13).

In August 2020, the FDA approved satralizumab for the treatment of AQP4-IgG–seropositive NMOSD. Satralizumab is a recombinant humanized IgG2 monoclonal antibody that binds to the IL-6 receptor, thereby inhibiting downstream IL-6 signaling. Compared to tocilizumab, satralizumab has a longer antibody half-life in circulation, offering extended therapeutic activity (14).

Previous phase III clinical trials have demonstrated the efficacy of satralizumab both as add-on therapy and as monotherapy in patients with NMOSD. In a study evaluating satralizumab as an add-on to baseline immunosuppressive therapy, 8 patients (20%) in the satralizumab group experienced protocol-defined relapses compared to 18 patients (43%) in the placebo group, yielding a hazard ratio of 0.38 (95% CI: 0.16–0.88) (15). Importantly, therapeutic benefit was observed in both AQP4-IgG–seropositive and –seronegative subgroups.

Another phase III trial evaluating satralizumab as monotherapy also reported a lower relapse rate and prolonged time to relapse in the treatment group compared to placebo (16). Furthermore, satralizumab has shown favorable safety and efficacy profiles in special populations such as adolescents and pregnant or postpartum women (17, 18).

These findings suggest that satralizumab offers a convenient treatment option, particularly for patients who cannot access hospital-based therapy. Additionally, injection-related reactions were generally mild and did not lead to treatment discontinuation or withdrawal in clinical studies.

In our study, we observed a rapid increase in the number of AE reports associated with satralizumab. According to the Weber effect—which suggests that AE reporting typically peaks within the first two years following a drug’s market approval and then declines (19)—the reporting rate would be expected to stabilize over time. However, the continued rise in reporting contradicts this trend, underscoring the need for ongoing pharmacovigilance and epidemiological surveillance.

Regarding the signal detection results, infection-related adverse events associated with satralizumab were highly significant and involved multiple organ systems as well as infectious complications (**Supplementary Table 3**). This finding aligns with a previous study on the infection risk in NMOSD patients, which reported a high incidence of infections following disease onset (20). Notably, in that study, most infections occurred several months after treatment initiation, but the authors concluded that these events were not directly attributable to immunotherapy.

The international consensus on the management of NMOSD also recommends timely vaccination prior to initiating biologic therapy whenever possible (21). But, these findings from the database are hypothesis-generating and should inform future analytic pharmacoepidemiology and cautious clinical awareness rather than definitive practice changes.

In addition to infections, previously reported adverse events such as elevated liver enzymes, neutropenia, hypersensitivity reactions, and dyslipidemia were also detected in our analysis1. Although signals for other events such as arthralgia and hypersensitivity were observed, they did not meet the predefined criteria for positive signals and were therefore excluded.

In addition, our analysis identified several adverse events that were not previously reported in clinical trials, including malignancies, cerebral infarction, and bone-related complications. Although no direct causal relationship between these events and satralizumab has been established, certain findings warrant further consideration. Notably, cerebral infarction was reported exclusively in male patients, which may be associated with a higher prevalence of comorbidities and an increased baseline risk of cardiovascular and cerebrovascular diseases in males. Glucocorticoids are commonly used as baseline therapy in NMOSD and were permitted in prior clinical trials evaluating satralizumab (22). Long-term glucocorticoid use is a known risk factor for osteoporosis and fractures, which may have contributed to the bone-related adverse events observed in our analysis.

However, due to the limitations of the FAERS database—including the lack of detailed patient medical history and comorbidity information—the potential association between satralizumab and malignancy requires further investigation through well-controlled epidemiological studies. Besides, interpretation of these “unexpected” AEs requires caution. NMOSD patients treated with satralizumab in routine practice may have advanced disease, prior treatment failure, and concomitant immunosuppression, all of which elevate baseline risks. Potential mechanistic links—such as infection-mediated prothrombotic states, corticosteroid-related bone loss, or surveillance bias for malignancy—may also contribute. However, FAERS/JADER cannot disentangle these confounders; signals should prompt further evaluation in controlled designs.

Our comparison between patients with serious and non-serious outcomes provides valuable insights for identifying high-risk individuals in clinical settings. Consistent with findings from previous clinical trials, infection-related adverse events were more frequently observed among patients with serious outcomes. This underscores the importance of infection prevention, early recognition of prodromal symptoms, and prompt clinical intervention. But due to the serious events are more likely to be reported promptly, whereas non-serious events may be delayed or underreported, thus informative delay/reporting bias can artifactually shorten observed induction times for serious AEs in spontaneous reporting data.

Moreover, serious outcomes tended to occur earlier in the treatment course, highlighting the critical need for close monitoring during the early phase of satralizumab therapy.

With respect to dosing regimens, analysis of real-world data indicated that the standard usage remains relatively safe. This usage is consistent with the approved product labeling for satralizumab (23). We therefore recommend strict adherence to the approved dosing schedule in clinical practice to minimize the risk of serious adverse events.

This study incorporated a large number of AEs reports related to satralizumab, providing valuable real-world evidence for post-marketing drug safety assessment. However, several limitations inherent to spontaneous reporting systems must be acknowledged. Approximately one-third of the reports were submitted by consumers, which may lead to underreporting, duplicate entries, and challenges in ensuring data quality. In NMOSD, relapses can present with weakness, pain, and sensory symptoms that may be misattributed to drug toxicity in patient-reported narratives. This raises a risk of outcome misclassification for disease-related neurological AEs. Signal interpretation for such PTs warrants additional caution, and stratified or sensitivity analyses by reporter type may be informative in future work. Besides, we acknowledge that requiring concordance across ROR/PRR/BCPNN/MGPS increases specificity but may miss rare AEs.

Additionally, the lack of detailed clinical information—such as patient medical history and comorbidities—precludes assessment of confounding factors. These databases lack reliable exposure denominators and detailed baseline risk information, the disproportionality metrics reflect reporting disproportionality rather than incidence or absolute risk.

Despite these limitations, pharmacovigilance studies remain essential for monitoring drug safety and detecting rare or unexpected adverse events that may not be captured during clinical trials.

## 5 Conclusion

This study identified several previously unreported adverse events associated with satralizumab that were not documented in earlier clinical trials, including fractures, cerebral infarction, and neuralgia. These findings provide important evidence to guide post-treatment monitoring and early detection strategies aimed at preventing serious outcomes.

In addition, adherence to the standardized dosing regimen may help reduce the risk of adverse events, further supporting the need for consistency in clinical practice. Overall, our results carry important implications for clinicians, patients, and healthcare policymakers in optimizing the safe use of satralizumab.

## Data Availability

The datasets presented in this study can be found in online repositories. The names of the repository/repositories and accession number(s) can be found in the article/**Supplementary Material**.

## Glossary

FDA: U.S. Food and Drug Administration
FAERS: FDA Adverse Event Reporting System
JADER: Japanese Adverse Drug Event Report
AE: adverse event
PS: primary suspect
ROR: Reporting Odds Ratio
PRR: Proportional Reporting Ratio
BCPNN: Bayesian Confidence Propagation Neural Network
MGPS: Multi-item Gamma Poisson Shrinker
CI: confidence intervals
IL-6: interleukin-6
NMOSD: neuromyelitis optica spectrum disorder
AQP4: aquaporin-4
PTs: preferred terms
