# Supplementary material for: Disproportionality analysis of satralizumab in FDA adverse event reporting system and Japanese adverse drug event report: a pharmacovigilance study
Source: Front Immunol. 2026 Jan 14;16:1647306. doi: 10.3389/fimmu.2025.1647306 (PMC12846964; doi:10.3389/fimmu.2025.1647306)

Supplementary table 1. Two-by-Two Contingency Table for Adverse Event Analysis.

|  | **Number of suspect adverse events** | **Number of other adverse events** | **Total** |
| --- | --- | --- | --- |
| Suspect drug | a | b | a+b |
| Other drug | c | d | c+d |
| Total | a+c | b+d | a+b+c+d |

Supplementary table 2. Comparison of Disproportionality Analysis Algorithms in Pharmacovigilance

| **Algorithms** | **Formula** | **Prerequisite** |
| --- | --- | --- |
| ROR | $ROR=\frac{(a/c)}{(b/d)}=\frac{\mathrm{ad}}{\mathrm{bc}}$ | lower limit of 95% CI>1, N≥3 |
|  | $95\%CI=e^{ln(ROR)\pm1.96\sqrt{(\frac{1}{a}+\frac{1}{b}+\frac{1}{c}+\frac{1}{d})}}$ |  |
| PRR | $PRR=\frac{a/{(a+b)}}{c/{(c+d)}}$ | PRR≥2, χ2≥4, N≥3 |
|  | $\chi2=\frac{{(ad-bc)}^{2}}{(a+b)(c+d)(a+c)(b+d)}$ |  |
| BCPNN | $IC=\log_{2}\frac{a(a+b+c+d)}{(a+c)(a+b)}$ | IC025>0, N≥3 |
|  | $E(IC)=\log_{2}\frac{(a+\gamma11)(a+b+c+d+\alpha)(a+b+c+d+\beta)}{(a+b+c+d+\gamma)(a+b+\alpha1)(a+c+\beta1)}$  95%CI=E(IC) ± 2V(IC)^0.5 |  |
|  | $V(IC)=\frac{1}{{(ln2)}^{2}}\left\{ \left[ \frac{(a+b+c+d)-\alpha+\gamma-\gamma11}{(\alpha+\gamma11)(1+a+b+c+d+\gamma)} \right]+\left[ \frac{(a+b+c+d)-(a+b)+\alpha-\alpha1}{(a+b+\alpha1)(1+a+b+c+d+\alpha)} \right]+\left[ \frac{(a+b+c+d)-(a+c)+\beta-\beta1}{(a+c+\beta1)(1+a+b+c+d+\beta)} \right] \right\}$ |  |
|  | $95\%CI=E(IC)\pm2\sqrt{v(IC)}$ |  |
| MGPS | $EBGM=\frac{a(a+b+c+d)}{(a+c)(a+b)}$ | EBGM05≥2,N≥3 |
|  | $95\%CI=e^{ln(EBGM)\pm1.96\sqrt{(\frac{1}{a}+\frac{1}{b}+\frac{1}{c}+\frac{1}{d}}}$ |  |

Note: a, number of reports containing both the suspect drug and the suspect adverse drug reaction; b, number of reports containing the suspect adverse drug reaction with other medications (except the drug of interest); c, number of reports containing the suspect drug with other adverse drug reactions (except the event of interest); d, number of reports containing other medications and other adverse drug reactions. ROR, reporting odds ratio; CI, confidence interval; N, the number of co-occurrences; PRR, proportional reporting ratio; χ2, chi-squared; BCPNN, Bayesian confidence propagation neural network; IC, information component; IC025, the lower limit of the 95% CI of the IC; E (IC), the IC expectations; V (IC), the variance of IC; MGPS, multi-item gamma Poisson shrinker; EBGM, empirical Bayesian geometric mean; EBGM05, the lower limit of the 95% CI of EBGM.

**Data algorithms**

This study employed four commonly used algorithms—ROR, PRR, BCPNN, and MGPS—to evaluate the association between the target drug and AEs. The ROR is a disproportionality measure that estimates the likelihood of a specific AE occurring in reports associated with a particular drug compared to all other drugs, using logistic regression analysis. The ROR algorithm considers the total number of reports and can be adjusted for potential confounding variables.Similar to ROR, the PRR is another algorithm used to detect potential drug-related safety signals. PRR identifies signals by comparing the proportion of reports of a specific AE associated with a particular drug to the proportion of the same AE associated with all other drugs.The BCPNN is an advanced Bayesian-based algorithm that evaluates the likelihood of a causal relationship between a drug and an AE. BCPNN is particularly effective in handling sparse data and generates fewer false-positive signals compared to PRR. This algorithm operates by propagating evidence strength through a network of interconnected nodes, continuously updating the probabilities of associations based on cumulative evidence.The MGPS is a shrinkage method similar to PRR or ROR, designed to minimize false-positive signals. MGPS applies a gamma distribution to observed counts and adjusts the estimates towards a central value (typically zero) to shrink signals. This approach highlights the most probable signals, providing focus for subsequent investigations.Each algorithm has distinct strengths and limitations, and their selection depends on the required balance between sensitivity and specificity for signal detection ^4^.

**Statistical Analysis**

A signal was considered to be present only when all four methods demonstrated statistical significance, defined as follows: lower bound of the 95% CI > 1 for ROR, > 0 for IC, > 2 for EBGM, and PRR ≥ 2. The analytical procedures are detailed in the Supplementary Materials. In addition, we used the JADER database as a comparator to assess the consistency of AE types across different populations. Demographic and clinical characteristics—including age, sex, body weight, AE type, and time to onset—were compared between serious and non-serious cases. Chi-square (χ²) tests were used to assess categorical variables such as sex and AE type, while the Mann–Whitney U test (M–W–U) was applied for continuous variables including age, body weight, and onset time. Based on the standard dosing regimen described in the drug label, logistic regression analysis was conducted to compare the safety profiles of alternative dosing regimens, with odds ratios (ORs) calculated accordingly.

Supplementary table 3.Safety signals for satralizumab-related adverse events with more than four counts are classified into four groups in FAERS

|  | PT | N | ROR(95%C) | PRR(95%CI) | EBGM(EBGM05) | IC(IC025) | Mean±SD | n |
| --- | --- | --- | --- | --- | --- | --- | --- | --- |
| Expected AE | Urinary Tract Infection | 72 | 10.41 ( 8.24 - 13.16 ) | 10.14 (9.92 - 10.37) | 10.14 ( 8.33 ) | 3.34 ( 3 ) | 234.70±267.66 | 34 |
|  | Pneumonia | 44 | 3.33 ( 2.47 - 4.48 ) | 3.29 (2.99 - 3.58) | 3.29 ( 2.56 ) | 1.72 ( 1.28 ) | 178.63±222.16 | 19 |
|  | Covid-19 | 43 | 5.76 ( 4.26 - 7.78 ) | 5.68 (5.38 - 5.97) | 5.68 ( 4.41 ) | 2.5 ( 2.07 ) | 211.74±228.37 | 23 |
|  | Hypoaesthesia | 24 | 3.78 ( 2.53 - 5.66 ) | 3.76 (3.36 - 4.16) | 3.76 ( 2.68 ) | 1.91 ( 1.33 ) | 56.6±61.11 | 10 |
|  | Sepsis | 23 | 4.91 ( 3.26 - 7.41 ) | 4.88 (4.47 - 5.29) | 4.88 ( 3.46 ) | 2.29 ( 1.69 ) | 182.38±207.27 | 13 |
|  | Cellulitis | 17 | 7.9 ( 4.91 - 12.74 ) | 7.86 (7.38 - 8.33) | 7.86 ( 5.27 ) | 2.97 ( 2.29 ) | 119.36±151.01 | 11 |
|  | Hepatic Function Abnormal | 15 | 9.86 ( 5.94 - 16.38 ) | 9.81 (9.3 - 10.31) | 9.8 ( 6.41 ) | 3.29 ( 2.57 ) | 74.85±77.36 | 13 |
|  | Lymphocyte Count Decreased | 15 | 19.82 ( 11.93 - 32.94 ) | 19.71 (19.21 - 20.22) | 19.7 ( 12.88 ) | 4.3 ( 3.58 ) | 196.71±275.39 | 14 |
|  | White Blood Cell Count Decreased | 15 | 3.28 ( 1.97 - 5.44 ) | 3.26 (2.76 - 3.77) | 3.26 ( 2.13 ) | 1.71 ( 0.98 ) | 200.43±269.62 | 7 |
|  | Herpes Zoster | 15 | 6.18 ( 3.72 - 10.27 ) | 6.15 (5.65 - 6.66) | 6.15 ( 4.02 ) | 2.62 ( 1.9 ) | 213.36±227.31 | 11 |
|  | Septic Shock | 14 | 7.91 ( 4.68 - 13.38 ) | 7.87 (7.35 - 8.4) | 7.87 ( 5.07 ) | 2.98 ( 2.23 ) | 216.88±240.49 | 8 |
|  | Cystitis | 13 | 9.49 ( 5.5 - 16.36 ) | 9.44 (8.9 - 9.99) | 9.44 ( 5.98 ) | 3.24 ( 2.47 ) | 409.86±321.84 | 7 |
|  | Pyelonephritis | 13 | 36.34 ( 21.06 - 62.7 ) | 36.16 (35.61 - 36.7) | 36.1 ( 22.87 ) | 5.17 ( 4.4 ) | 192.75±188.48 | 8 |
|  | Pneumonia Aspiration | 10 | 9.61 ( 5.16 - 17.88 ) | 9.57 (8.95 - 10.19) | 9.57 ( 5.69 ) | 3.26 ( 2.39 ) | 116.63±102.50 | 8 |
|  | Liver Disorder | 10 | 5.43 ( 2.92 - 10.1 ) | 5.41 (4.79 - 6.03) | 5.41 ( 3.22 ) | 2.44 ( 1.56 ) | 78±143.65 | 6 |
|  | White Blood Cell Count Increased | 8 | 4.82 ( 2.41 - 9.65 ) | 4.81 (4.12 - 5.5) | 4.81 ( 2.69 ) | 2.26 ( 1.3 ) | 359.5±465.98 | 2 |
|  | Hepatic Steatosis | 8 | 10.28 ( 5.13 - 20.58 ) | 10.25 (9.56 - 10.94) | 10.25 ( 5.73 ) | 3.36 ( 2.39 ) | 237±243.24 | 2 |
|  | Blood Cholesterol Increased | 8 | 4.11 ( 2.05 - 8.23 ) | 4.1 (3.41 - 4.79) | 4.1 ( 2.29 ) | 2.04 ( 1.07 ) | 54±37.89 | 5 |
|  | Atypical Mycobacterial Infection | 7 | 134.43 ( 63.87 - 282.93 ) | 134.06 (133.32 - 134.8) | 133.24 ( 71.48 ) | 7.06 ( 6.03 ) | 273.29±214.25 | 7 |
|  | Osteomyelitis | 6 | 7.71 ( 3.46 - 17.19 ) | 7.7 (6.9 - 8.5) | 7.69 ( 3.94 ) | 2.94 ( 1.85 ) | 193.67±102.55 | 3 |
|  | Dyslipidaemia | 5 | 23.12 ( 9.61 - 55.63 ) | 23.08 (22.2 - 23.96) | 23.06 ( 11.06 ) | 4.53 ( 3.35 ) | 97±107.52 | 4 |
|  | Melaena | 5 | 5.29 ( 2.2 - 12.71 ) | 5.28 (4.4 - 6.15) | 5.28 ( 2.53 ) | 2.4 ( 1.22 ) | 63±49.50 | 2 |
|  | Covid-19 Pneumonia | 5 | 9.93 ( 4.13 - 23.9 ) | 9.92 (9.04 - 10.79) | 9.91 ( 4.76 ) | 3.31 ( 2.13 ) | 193.6±246.99 | 5 |
|  | Gamma-Glutamyltransferase Increased | 5 | 5.16 ( 2.15 - 12.41 ) | 5.15 (4.28 - 6.03) | 5.15 ( 2.47 ) | 2.36 ( 1.19 ) | 103.33±124.27 | 3 |
|  | Kidney Infection | 5 | 6.11 ( 2.54 - 14.69 ) | 6.1 (5.22 - 6.97) | 6.1 ( 2.93 ) | 2.61 ( 1.43 ) | 396±0 | 1 |
|  | Pneumonia Bacterial | 5 | 13.78 ( 5.73 - 33.14 ) | 13.75 (12.88 - 14.63) | 13.74 ( 6.59 ) | 3.78 ( 2.6 ) | 211.2±161.81 | 5 |
|  | Hypogammaglobulinaemia | 5 | 21.2 ( 8.81 - 50.99 ) | 21.16 (20.28 - 22.03) | 21.14 ( 10.14 ) | 4.4 ( 3.22 ) | 97±49.50 | 2 |
|  | Coronavirus Infection | 4 | 14.02 ( 5.26 - 37.4 ) | 14 (13.02 - 14.98) | 13.99 ( 6.16 ) | 3.81 ( 2.51 ) | 256±265.87 | 2 |
|  | Blood Triglycerides Increased | 4 | 5.3 ( 1.99 - 14.14 ) | 5.3 (4.32 - 6.27) | 5.29 ( 2.33 ) | 2.4 ( 1.11 ) | 177.33±105.20 | 3 |
|  | Decubitus Ulcer | 4 | 11.47 ( 4.3 - 30.59 ) | 11.45 (10.47 - 12.43) | 11.45 ( 5.04 ) | 3.52 ( 2.22 ) | 212.67±181.71 | 3 |
|  | Liver Function Test Increased | 4 | 4.77 ( 1.79 - 12.71 ) | 4.76 (3.78 - 5.74) | 4.76 ( 2.09 ) | 2.25 ( 0.96 ) | 49±49.50 | 2 |
|  | Appendicitis | 4 | 10.55 ( 3.96 - 28.15 ) | 10.54 (9.56 - 11.52) | 10.53 ( 4.64 ) | 3.4 ( 2.1 ) | NA | 0 |
| disease-related AE | Neuromyelitis Optica Spectrum Disorder | 128 | 3650.2 ( 3015.73 - 4418.16 ) | 3465.36 (3465.17 - 3465.54) | 2983.88 ( 2543.29 ) | 11.54 ( 11.27 ) | 239.67±243.74 | 48 |
|  | Muscular Weakness | 16 | 3.34 ( 2.05 - 5.47 ) | 3.33 (2.84 - 3.82) | 3.33 ( 2.21 ) | 1.74 ( 1.03 ) | 238.5±375.25 | 6 |
|  | Optic Neuritis | 12 | 30.94 ( 17.54 - 54.58 ) | 30.8 (30.24 - 31.37) | 30.76 ( 19.13 ) | 4.94 ( 4.14 ) | 241.33±238.39 | 6 |
|  | Myelitis | 7 | 112.21 ( 53.34 - 236.07 ) | 111.9 (111.16 - 112.64) | 111.33 ( 59.75 ) | 6.8 ( 5.77 ) | 168±105.13 | 3 |
|  | Visual Acuity Reduced | 7 | 4.73 ( 2.25 - 9.93 ) | 4.72 (3.98 - 5.46) | 4.72 ( 2.54 ) | 2.24 ( 1.22 ) | 171.5±91.37 | 4 |
|  | Blindness | 7 | 4.17 ( 1.99 - 8.76 ) | 4.16 (3.42 - 4.9) | 4.16 ( 2.24 ) | 2.06 ( 1.04 ) | 112±0 | 1 |
|  | Neuralgia | 7 | 6.97 ( 3.32 - 14.64 ) | 6.96 (6.22 - 7.7) | 6.95 ( 3.74 ) | 2.8 ( 1.78 ) | 5±0 | 1 |
|  | Sensory Disturbance | 5 | 7.14 ( 2.97 - 17.16 ) | 7.12 (6.25 - 8) | 7.12 ( 3.42 ) | 2.83 ( 1.65 ) | 15±0 | 1 |
|  | Visual Field Defect | 4 | 12.89 ( 4.83 - 34.39 ) | 12.87 (11.9 - 13.85) | 12.87 ( 5.66 ) | 3.69 ( 2.39 ) | 158±45.25 | 2 |
|  | Monoparesis | 4 | 51.35 ( 19.23 - 137.08 ) | 51.27 (50.29 - 52.25) | 51.15 ( 22.49 ) | 5.68 ( 4.38 ) | 441±0 | 1 |
|  | Hemiparesis | 4 | 5.45 ( 2.04 - 14.53 ) | 5.44 (4.46 - 6.42) | 5.44 ( 2.4 ) | 2.44 ( 1.15 ) | NA | 0 |
| Comorbidities-related AEs | Fracture | 10 | 12.55 ( 6.74 - 23.36 ) | 12.51 (11.89 - 13.12) | 12.5 ( 7.43 ) | 3.64 ( 2.77 ) | 107±122.05 | 3 |
|  | Compression Fracture | 8 | 43.76 ( 21.84 - 87.66 ) | 43.63 (42.93 - 44.32) | 43.54 ( 24.34 ) | 5.44 ( 4.48 ) | 336±105.59 | 3 |
|  | Osteoporosis | 7 | 3.99 ( 1.9 - 8.38 ) | 3.98 (3.24 - 4.72) | 3.98 ( 2.14 ) | 1.99 ( 0.97 ) | 170.5±226.98 | 2 |
|  | Spinal Compression Fracture | 6 | 15.44 ( 6.93 - 34.41 ) | 15.41 (14.61 - 16.21) | 15.4 ( 7.87 ) | 3.94 ( 2.85 ) | 32±43.84 | 2 |
|  | Osteonecrosis | 9 | 6.12 ( 3.18 - 11.78 ) | 6.1 (5.45 - 6.76) | 6.1 ( 3.53 ) | 2.61 ( 1.69 ) | 340.67±248.29 | 3 |
| Unexpected | Cerebral Infarction | 6 | 5.74 ( 2.57 - 12.78 ) | 5.72 (4.92 - 6.52) | 5.72 ( 2.93 ) | 2.52 ( 1.42 ) | 132±124.77 | 4 |
|  | Ovarian Cancer | 5 | 12.44 ( 5.17 - 29.93 ) | 12.42 (11.55 - 13.3) | 12.41 ( 5.96 ) | 3.63 ( 2.45 ) | 434±299.41 | 3 |
|  | Walking Distance Test Abnormal | 4 | 250.03 ( 93.24 - 670.52 ) | 249.64 (248.65 - 250.62) | 246.78 ( 108.1 ) | 7.95 ( 6.65 ) | 18.33±2.89 | 3 |

Supplementary table 4. Safety signals for satralizumab-related adverse events with more than four counts are classified into four groups in JADER

| PT | a | ROR(95%CI) | PRR(95%CI) | EBGM | IC |
| --- | --- | --- | --- | --- | --- |
| Neuromyelitis optica spectrum disorder* | 53 | 1777.49 (1274.7 - 2478.59) | 1526.41 (1097.43 - 2123.07) | 1122.18 (804.75) | 10.13 (8.45) |
| Urinary tract infection* | 21 | 42.67 (27.42 - 66.4) | 40.34 (25.94 - 62.73) | 39.97 (25.68) | 5.32 (3.65) |
| Pneumonia* | 17 | 3.77 (2.32 - 6.14) | 3.65 (2.24 - 5.93) | 3.64 (2.24) | 1.87 (0.19) |
| Lymphocyte count decreased* | 17 | 26.28 (16.13 - 42.8) | 25.13 (15.44 - 40.91) | 24.99 (15.34) | 4.64 (2.97) |
| Cellulitis* | 12 | 17.74 (9.97 - 31.56) | 17.2 (9.67 - 30.6) | 17.14 (9.63) | 4.1 (2.43) |
| Sepsis* | 11 | 7.15 (3.92 - 13.02) | 6.97 (3.82 - 12.69) | 6.96 (3.82) | 2.8 (1.13) |
| Pneumonia aspiration* | 8 | 9.61 (4.76 - 19.37) | 9.42 (4.67 - 19) | 9.41 (4.66) | 3.23 (1.56) |
| Septic shock* | 8 | 11.93 (5.92 - 24.06) | 11.7 (5.8 - 23.59) | 11.67 (5.79) | 3.54 (1.87) |
| White blood cell count decreased* | 7 | 1.9 (0.9 - 4.01) | 1.88 (0.89 - 3.98) | 1.88 (0.89) | 0.91 (-0.76) |
| Pyelonephritis* | 7 | 21.26 (10.05 - 45) | 20.88 (9.87 - 44.19) | 20.79 (9.82) | 4.38 (2.7) |
| Escherichia urinary tract infection | 6 | 397.13 (171.02 - 922.2) | 390.8 (168.31 - 907.39) | 357.86 (154.11) | 8.48 (6.78) |
| Pneumonia bacterial* | 6 | 10.07 (4.49 - 22.58) | 9.92 (4.43 - 22.25) | 9.9 (4.42) | 3.31 (1.63) |
| COVID-19* | 5 | 6.75 (2.79 - 16.33) | 6.68 (2.76 - 16.14) | 6.67 (2.76) | 2.74 (1.06) |
| Pancytopenia | 4 | 2.17 (0.81 - 5.82) | 2.16 (0.81 - 5.78) | 2.16 (0.81) | 1.11 (-0.56) |
| Herpes zoster* | 4 | 5.27 (1.97 - 14.13) | 5.23 (1.95 - 14) | 5.22 (1.95) | 2.38 (0.71) |
| Anaemia | 4 | 1.05 (0.39 - 2.81) | 1.05 (0.39 - 2.81) | 1.05 (0.39) | 0.07 (-1.61) |

*signal detected

Supplementary figure 1. The Volcano Map to Visualize the Differences in AE severity in male group.


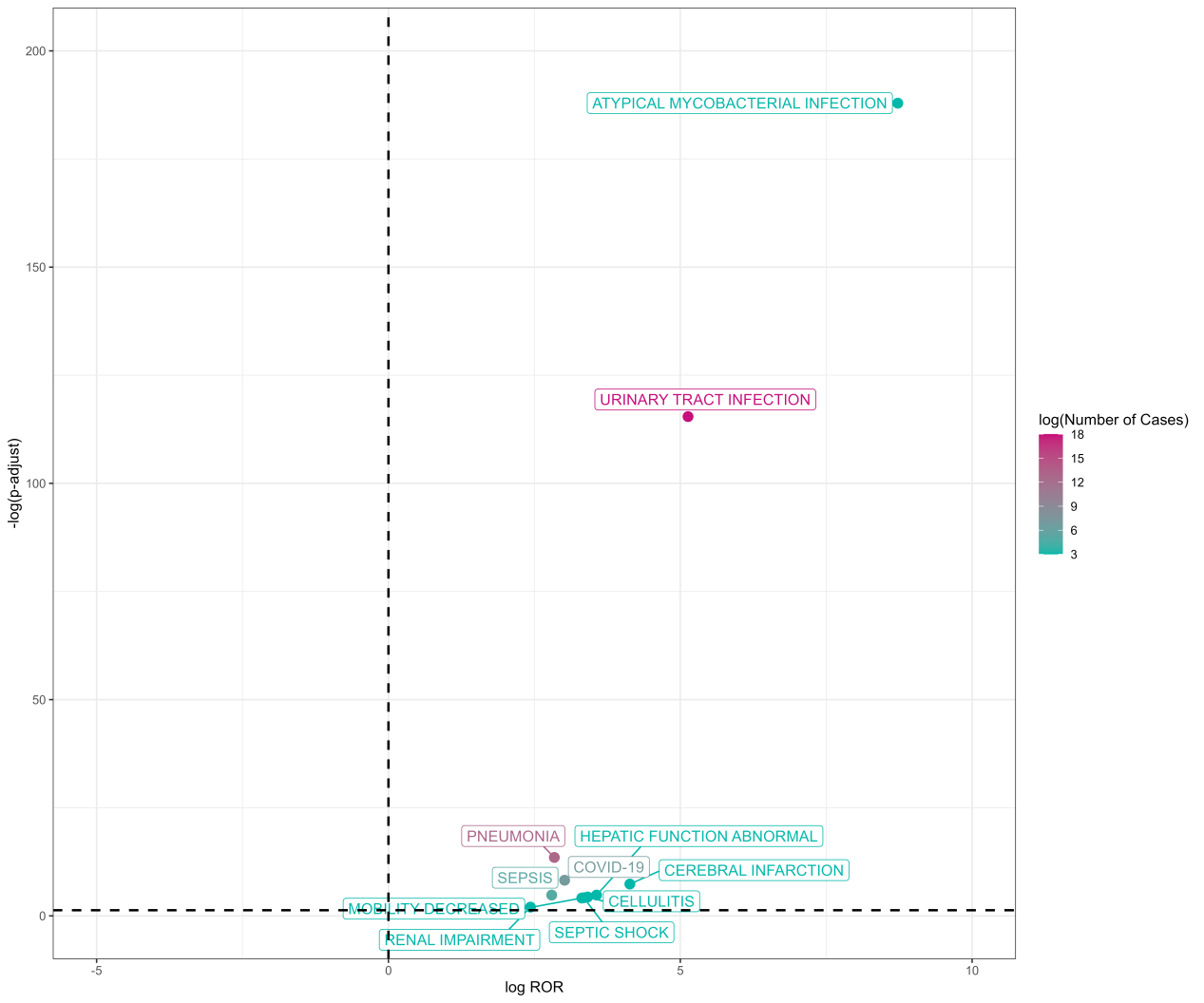


Supplementary figure 2. The Volcano Map to Visualize the Differences in AE severity in female group.


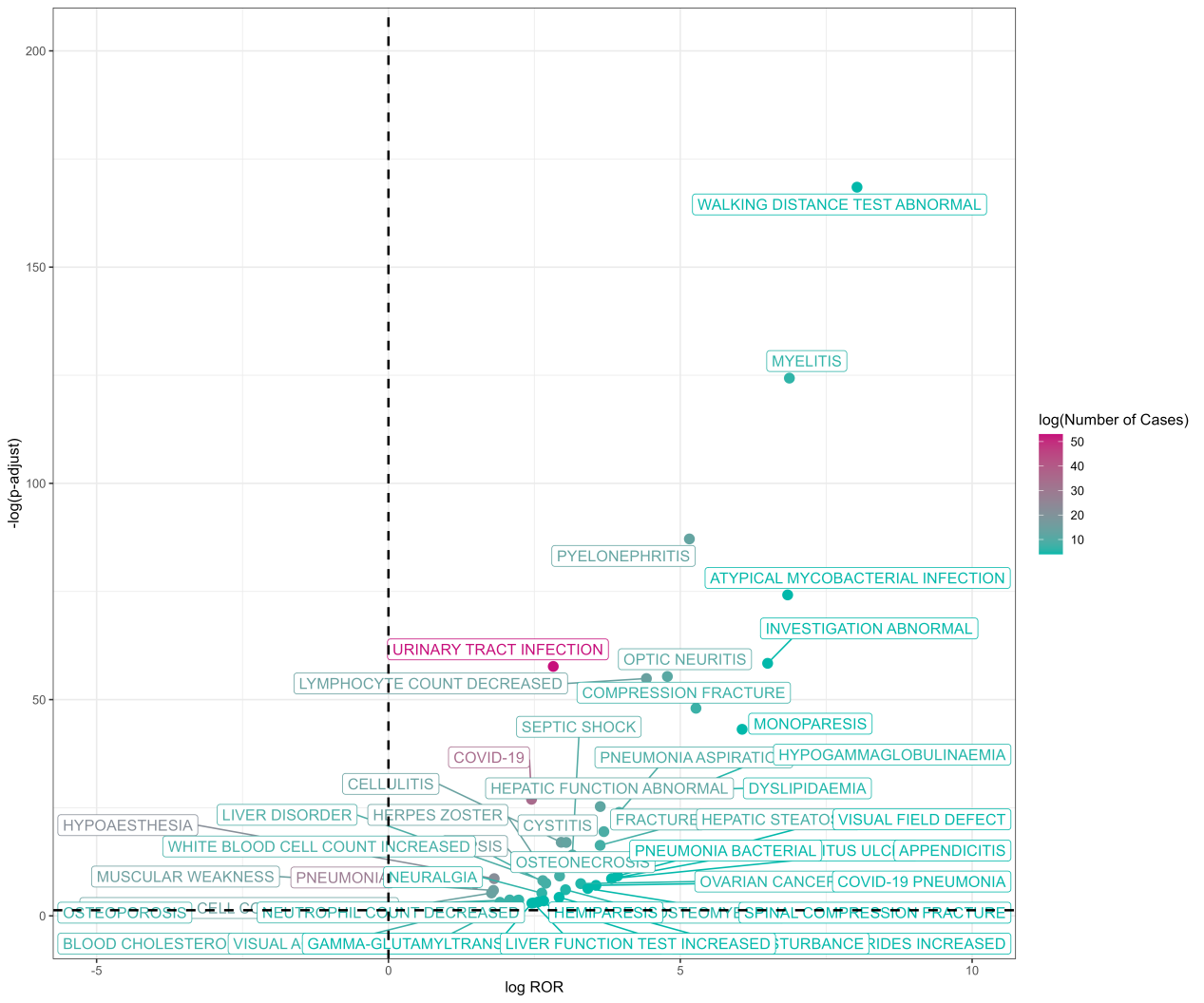

Supplement: Supplementary file 1 [file DataSheet1.docx]
